# Reduced Heart Rate Variability in Social Anxiety Disorder: Associations with Gender and Symptom Severity

**DOI:** 10.1371/journal.pone.0070468

**Published:** 2013-07-30

**Authors:** Gail A. Alvares, Daniel S. Quintana, Andrew H. Kemp, Anita Van Zwieten, Bernard W. Balleine, Ian B. Hickie, Adam J. Guastella

**Affiliations:** 1 Brain & Mind Research Institute, The University of Sydney, Sydney, New South Wales, Australia; 2 School of Psychology, The University of Sydney, Sydney, New South Wales, Australia; 3 Hospital Universitário, University of São Paulo, São Paulo, Brazil; Institute of Psychiatry at the Federal University of Rio de Janeiro, Brazil

## Abstract

**Background:**

Polyvagal theory emphasizes that autonomic nervous system functioning plays a key role in social behavior and emotion. The theory predicts that psychiatric disorders of social dysfunction are associated with reduced heart rate variability, an index of autonomic control, as well as social inhibition and avoidance. The purpose of this study was to examine whether heart rate variability was reduced in treatment-seeking patients diagnosed with social anxiety disorder, a disorder characterized by social fear and avoidance.

**Methods:**

Social anxiety patients (*n* = 53) were recruited prior to receiving psychological therapy. Healthy volunteers were recruited through the University of Sydney and the general community and were matched by gender and age (*n* = 53). Heart rate variability was assessed during a five-minute recording at rest, with participants completing a range of self-report clinical symptom measures.

**Results:**

Compared to controls, participants with social anxiety exhibited significant reductions across a number of heart rate variability measures. Reductions in heart rate variability were observed in females with social anxiety, compared to female controls, and in patients taking psychotropic medication compared to non-medicated patients. Finally, within the clinical group, we observed significant associations between reduced heart rate variability and increased social interaction anxiety, psychological distress, and harmful alcohol use.

**Conclusions:**

The results of this study confirm that social anxiety disorder is associated with reduced heart rate variability. Resting state heart rate variability may therefore be considered a marker for social approach-related motivation and capacity for social engagement. Additionally, heart rate variability may provide a useful biomarker to explain underlying difficulties with social approach, impaired stress regulation, and behavioral inhibition, especially in disorders associated with significant impairments in these domains.

## Introduction

Epidemiological evidence suggests that anxiety disorders place an individual at higher risk of fatal cardiovascular diseases (CVD), including coronary heart disease and sudden cardiac death [1]. It has been suggested that dysregulated autonomic nervous system (ANS) activity [2] may underlie this three-fold increase in risk for CVD [3] in anxiety. With an early age of onset and high lifetime frequency, social anxiety disorder is placed third only in prevalence to depression and alcohol dependence [4]. Despite the known role of autonomic arousal as a maintenance feature within social anxiety [5], it represents an under-evaluated disorder with respect to dysregulation in ANS functioning, in particular autonomic cardiac control.

Polyvagal theory is a biobehavioral model that links ANS functioning and social engagement. It proposes that the ANS evolved specifically in mammals to modulate an individual’s affective experience and subsequent social behavior [6]. In particular, the theory emphasizes the role of the vagus nerve, the primary nerve of the parasympathetic nervous system, in promoting engagement, or disengagement, with an individual’s social environment. Such cardiac vagal control inhibits sympathetic activity to promote prosocial behaviour and regulate emotion [7].

Heart rate variability (HRV), an index of beat-to-beat changes in heart rate, provides a non-invasive measure of ANS activity [8]. Optimal cardiac health is characterized by increased variability, with lower HRV linked to CVD and mortality [9]. As predicted by Polyvagal theory, clinical conditions associated with decreases in social functioning and capacity for social engagement are reflected in decreased regulation of ANS function, or reduced HRV. This then supports the proposal that social anxiety disorder, a prototypical disorder of social avoidance and disengagement, will also be associated with reductions in autonomic cardiac control. Further, as predicted by the theory, these reductions in autonomic control may be associated with social inhibition, avoidance, and fear. However, no study to date has specifically examined whether hypothesized reductions in HRV in social anxiety are associated with measures of symptom severity or social functioning.

Whilst reduced HRV has been demonstrated broadly across anxiety disorders in large cohort studies [10], there has been limited evaluation of social anxiety disorder specifically. Previous research has demonstrated significant reductions in HRV in geriatric samples with social anxiety [11] and in patients with a number of comorbid anxiety disorders, including social anxiety [12]. More recent evidence suggests that, in patients with social anxiety, changes in HRV over time predicts response to psychological treatment [13].

A number of additional factors may contribute to dysregulated autonomic cardiac control in social anxiety. In particular, negative effects of psychotropic medications on HRV has been previously discussed in [10]. Epidemiological evidence also highlights gender differences in HRV, with females exhibiting greater parasympathetic activity, whilst males have greater sympathetic activity, at rest (reviewed in [14], [15]). Given higher prevalence rates of anxiety disorders in females [4], and potential cardioprotective effects of estrogen [16], there may be gender differences in HRV that are particularly pronounced in social anxiety disorder.

Thus, the objective of the present study was to investigate HRV in a community-based sample of treatment-seeking patients with primary social anxiety disorder. We hypothesized that individuals with social anxiety would exhibit reduced resting-state HRV, relative to a control group. We also predicted that gender may moderate these differences between groups. Further, we hypothesized that reduced resting-state HRV may be associated with clinical measures of symptom severity and functioning.

## Methods

### Ethics Statement

This study was approved by the University of Sydney’s Human Research Ethics Committee (12810), with all participants providing written informed consent.

### Participants

Participants in the social anxiety group (SAD) were recruited through the Anxiety Clinic at the Brain & Mind Research Institute (*n* = 80) between May 2011 and October 2012. Individuals were seeking treatment for specific social anxiety group treatment program, self-referred or referred by a mental health professional. Exclusion included presence of any medical conditions that may influence cardiovascular functioning (e.g. diabetes, deep vein thrombosis, immune disorders), missing interbeat interval (IBI) data, previous participation in social anxiety group treatment, and social anxiety not meeting criteria for a primary disorder, leaving a final sample of *n* = 53.

Of this sample, 83% of SAD participants (*n = *44) were comorbid with at least one other Axis I disorder. Six participants met criteria for substance dependence (alcohol or cannabis), with two casual cannabis users and nine regular smokers (defined as daily cigarette consumption); 55% of participants (*n* = 29) were taking at least one or more psychotropic medications (combinations of antidepressants, mood stabilizers, antipsychotics, and benzodiazepines). Other medications reported being used on the day of testing included oral contraceptives, health supplements (e.g. fish oil, St Johns Wort), pain relievers (e.g. paracetemol), substance withdrawal treatment (e.g. Disulfiram), asthma treatment (e.g. Ventolin), and antibiotics.

Control participants were students from the University of Sydney or individuals recruited from the general community using newspaper advertisements. Exclusion criteria included current or history of any psychiatric illness, current use of psychotropic medications, current medical conditions that could influence cardiovascular functioning, and equipment problems. We selected a sample of these participants, individually matching for gender, and matched groups for age, leaving a final sample of *n* = 53. Medications used in the control group included oral contraceptives, pain relievers, asthma treatments, and heartburn medication, with three casual cannabis users and four regular smokers.

### Materials

Clinical participants met criteria for a primary diagnosis of Social Anxiety Disorder as assessed by authors GAA, AJG, or trained research psychologists, using the Anxiety Disorders Interview Schedule for Adults [17] and DSM-IV-TR criteria [18]. Control participants were assessed by a brief self-report interview to ensure no current or history of any mental illness, based on the screening tool for the Structured Clinical Interview for DSM-IV-TR [19].

Self-report questionnaires completed by all participants included the Depression, Anxiety, Stress scale (DASS, 21 items, three subscales [20]), Social Phobia Anxiety Inventory 23-item version (SPAI [21]), Alcohol Use Disorder Identification Test (AUDIT [22]), and the International Physical Activity Questionnaire [23]. The DASS measures the tripartite negative emotional states of depression, anxiety, and stress experienced in the last week, with high internal consistency and reliability [24]. The shorter version of the SPAI utilized in this study was a brief measure of cognitive, behavioral, and somatic symptoms associated with social anxiety, which also differentiates symptoms associated with agoraphobia. This version has good discriminability between nonclinical students and individuals with social anxiety, compared to the original 45-item version, and acceptable convergent validity with other measures of social anxiety [25]. The AUDIT was utilized as a screening tool for the identification of potential alcohol abuse or dependence. The consumption subscale utilizes the first three items of the AUDIT that reliably identifies hazardous drinking levels, more indicative of a possible active alcohol use disorder. Additionally, the IPAQ short-form was used to measure overall energy expenditure, in minutes of metabolic expenditure (MET), based on levels of physical activity intensity [23].

Additional questionnaires obtained from the social anxiety group that measured symptoms of social anxiety were the Social Interaction Anxiety Scale and Social Phobia Scale (SIAS and SPS, respectively [26]), and the Liebowitz Social Anxiety Scale (LSAS [27]), whilst the Kessler psychological distress scale (K10 [28]) was included as a brief screening tool for the presence of mental health concerns, assessing levels of anxiety and depressive symptoms. The SIAS assesses anxiety and distress associated with social interactions, whilst the companion SPS measures anxiety when being observed, or anticipation of being observed, in social situations. The LSAS more specifically asks individuals to differentially rate their fear and avoidance of situations involving performance (e.g. public speaking), being observed, drinking or eating in public, or general social interactions. Missing values within questionnaires (<20%) were replaced with the mean for that scale or subscale, an imputation strategy that is valid for smaller percentages of missing data [29]. Missing questionnaires, or questionnaires with more than 20% of missing values, were excluded listwise from analyses.

IBIs were measured for 5 minutes via the Polar RS800CX (Polar Electro Oy, Kempele, Finland; 1000 Hz) heart rate monitor. This system wirelessly collects heart rate data from a two-lead chest strap. Whilst there has been debate over the validity of measuring R-R intervals with Polar monitors [30], [31], mobile devices have been shown to provide accurate and reliable data on par with that collected in electrocardiogram recordings [32]. In particular, Weippert and colleagues demonstrated excellent agreement between Polar monitors and traditional ECGs, when comparing simultaneous R-R interval recordings between the two devices [28].

### Procedure

After obtaining consent, all participants completed a standardized research assessment, with SAD participants completing the assessment in the week before or after the first group therapy session they were enrolled in. Both groups completed basic demographic questions, including questions about smoking patterns, drug and alcohol consumption, and medication use, as well as other self-report measures before heart rate measurements were collected. The SAD group completed the additional questionnaires no more than two weeks after this assessment. Height and weight were also measured to calculate body mass index (BMI). Both groups were instructed to abstain from caffeine, alcohol, and illicit substances on the day of, and from food and drink (other than water) for two hours prior to, testing.

Following an initial resting period lasting between 2 and 3 minutes, 5-minute IBI recordings were made during a relaxed, seated resting-state. Participants were instructed to sit quietly with their eyes open for the entire duration of recording. No instructions were made about breathing rate; participants were allowed to breathe spontaneously during the recording period. Although controlling for respiration rate has been subject to some discussion recently, it has been argued that respiration rate does not affect short-term, resting state HRV recordings, as long as a sufficient acclimatization period occurs prior to recording [33].

### Physiological Data Processing

Raw heart rate data was extracted as a text file from Polar ProTrainer (version 5, Polar Electro Oy, Kempele, Finland) and imported into Kubios (version 2.0, 2008, Biosignal Analysis and Medical Imaging Group, University of Kuopio, Finland, MATLAB). All 5 minute samples were initially processed with an automatic filter to remove potential artifacts. Artifact removal was confirmed via visual inspection after processing by authors GAA and DSQ. Kubios was then used to calculate time, frequency, and non-linear HRV measures. IBIs were calculated and transformed into beats per minute for an estimate of mean heart rate (MHR). The two time-domain measures calculated using IBIs included the standard deviation of all R-R intervals (SDNN) and the square root of the mean-squared differences between successive R-R intervals (RMSSD). The frequency domain measures were calculated as absolute powers of the power spectrum density in the high frequency (HF; 0.15–0.4 Hz) and low frequency (LF; 0.04–0.15 Hz) bands using the Fast Fourier transform. The HF component of the power spectrum reflects parasympathetic activity, whilst the LF component is argued to reflect a mixture of vagal and sympathetic influences [8]. The standard deviation of the Poincaré plot perpendicular to the line of identity (PCSD1) is a non-linear measure plotting each R-R interval as a function of the next R-R interval, with the standard deviation of this plot representing parasympathetic activity caused by respiratory sinus arrhythmia. Detrended fluctuation analysis (short-fluctuation slope, DFAα1) is a second non-linear variable that measures the correlation between successive R-R intervals; lower values indicate increased variability.

### Statistical Analyses

Analyses were conducted in SPSS (version 20) with significance set at *p*<.05. Independent samples *t*-tests compared differences between the social anxiety group and controls on baseline measures. Where Levene’s test for equality of variances was significant, adjusted *p-*values are reported. Chi-squared tests were conducted on gender, smoking status, and medication use. Tests were conducted to ensure that underlying assumptions for between-group analyses were not violated. According to the Kolmogorov-Smirnov statistic, all HRV measures, except for DFAα1 were not normally distributed with kurtosis statistics indicative of significant positive skew. Therefore a logarithmic transformation to base 10 was applied after which all values were normally distributed with no evidence of significant skew. All HRV variables were then tested within multivariate analyses of variance, with Group (SAD vs. control) and Gender as between-group variables. Cohen’s *d* was used as an effect size estimate between groups, with effects sizes of *d* = .2 interpreted as small, *d* = .5 medium, and *d* = .8 large [34]. All correlational analyses were conducted with Pearson correlations, two-tailed. The correlation coefficient was interpreted as *r* = .5 large, *r* = .3 medium, and *r* = .10 small [34]. Analyses aimed to investigate (a) whether HRV differed between social anxiety and controls; (b) whether gender moderated a main effect of group; (c) the impact of comorbidity and medication use; and (d) associations with clinical measures of self-reported symptoms.

## Results

### Participant Characteristics

Table 1 presents participant characteristics for the SAD and control groups, separated by gender. Of note, the SAD group had a significantly higher BMI than controls (*F*(1, 99) = 5.46, *p* = .02). Although decreases in HRV have been associated with age [35], [36], mixed evidence exists about relationships with BMI or physical fitness [36], [37]. In terms of gender, there were no significant differences between males and females in age, BMI, or smoking use. However, females reported slightly higher anxiety (as measured by the DASS-A;, (*F*(1, 102) = 3.13, *p* = .08), and significantly higher stress (DASS-S; *F*(1, 102) = 4.33, *p* = .04), and specific social anxiety (SPAI; *F*(1, 100) = 4.63, *p* = .03).

**Table 1:** Participant characteristics.

	SAD		Control		*p* values		
	Male	Female	Male	Female			
	(n = 32)	(n = 21)	(n = 32)	(n = 21)	Group	Gender	Group×Gender
**Age**	25.66 (5.77)	23.57 (6.09)	25.44 (8.30)	22.95 (6.45)	.76	.10	.88
**BMI^a^**	24.19 (3.46)	25.63 (6.12)	23.95 (3.12)	22.13 (3.18)	.02	.81	.04
**SPAI^b^**	53.76 (14.79)	61.17 (15.02)	14.53 (10.15)	17.90 (9.16)	<.001	.03	.42
**DASS-D**	21.03 (8.05)	23.81 (9.84)	5.62 (6.23)	5.27 (4.31)	<.001	.41	.29
**DASS-A**	16.00 (8.25)	20.76 (9.87)	4.69 (6.52)	5.32 (5.61)	<.001	.08	.18
**DASS-S**	20.75 (8.00)	24.19 (10.99)	6.94 (7.55)	10.48 (7.45)	<.001	.04	.97
**AUDIT-C^c^**	4.06 (3.58)	3.14 (2.43)	5.00 (3.01)	3.05 (2.56)	.49	.02	.39
**MET^d^**	3385.93 (3015.07)	2367.71 (3785.12)	3189.60 (1861.31)	4093.88 (4003.13)	.25	.93	.14
**Smoke (yes/no)**	6/32	3/21	4/32	1/21	.26	.36	–

*Note.* Numbers depict means (and standard deviations) or numbers in each category. Significance values taken from main and interaction ANOVA tests or chi-squared tests. BMI = Body Mass Index in kg/m^2^; SPAI = Social Phobia Anxiety Inventory, 23-item version, total score summing social anxiety and agoraphobia subscales; DASS = Depression, Anxiety, Stress scales, 21-item version; AUDIT-C = Alcohol Use Disorder Identification Test, consumption subscale; MET = estimate of metabolic energy expenditure in minutes per week. ^a^SAD group *n* = 51 ^b^SAD group *n* = 50 ^c^SAD group *n* = 52 ^d^SAD group *n* = 48, Control group *n* = 51.

In the SAD group, females reported significantly higher social interaction anxiety compared to males (SIAS; *t*(47) = 2.01, *p* = .05), with a trend towards higher specific social anxiety (SPAI social phobia subscale; *t*(49) = 1.92, *p* = .06) and significantly higher psychological distress (k10; *t*(47) = 2.39, *p* = .02). There were less non-medicated (n = 6) than medicated (n = 18) females, compared to males (medicated n = 14, non-medicated n = 15; Χ^2^ (1) = 3.92, *p* = .05). There were no significant differences between males and females in number of comorbid disorders, and no significant correlations between age, BMI, and any of the symptom measures; all *p*-values >.05.

### Between Group Differences in HRV

Overall multivariate ANOVA, with Group (SAD, Control) and Gender (Male, Female) as between-subjects variables, revealed significant differences across the log-transformed HRV measures in the SAD group, relative to controls (*F*(6, 97) = 2.58, *p* = .02), as well as significant differences between females compared to males (*F*(6, 97) = 7.11, *p*<.001). Although there was no overall significant interaction between group and gender (*F*(6, 97) = 1.30, *p* = .26), given our planned comparisons, we continued to follow-up on these main effects of group and gender.

**Figure 1:**
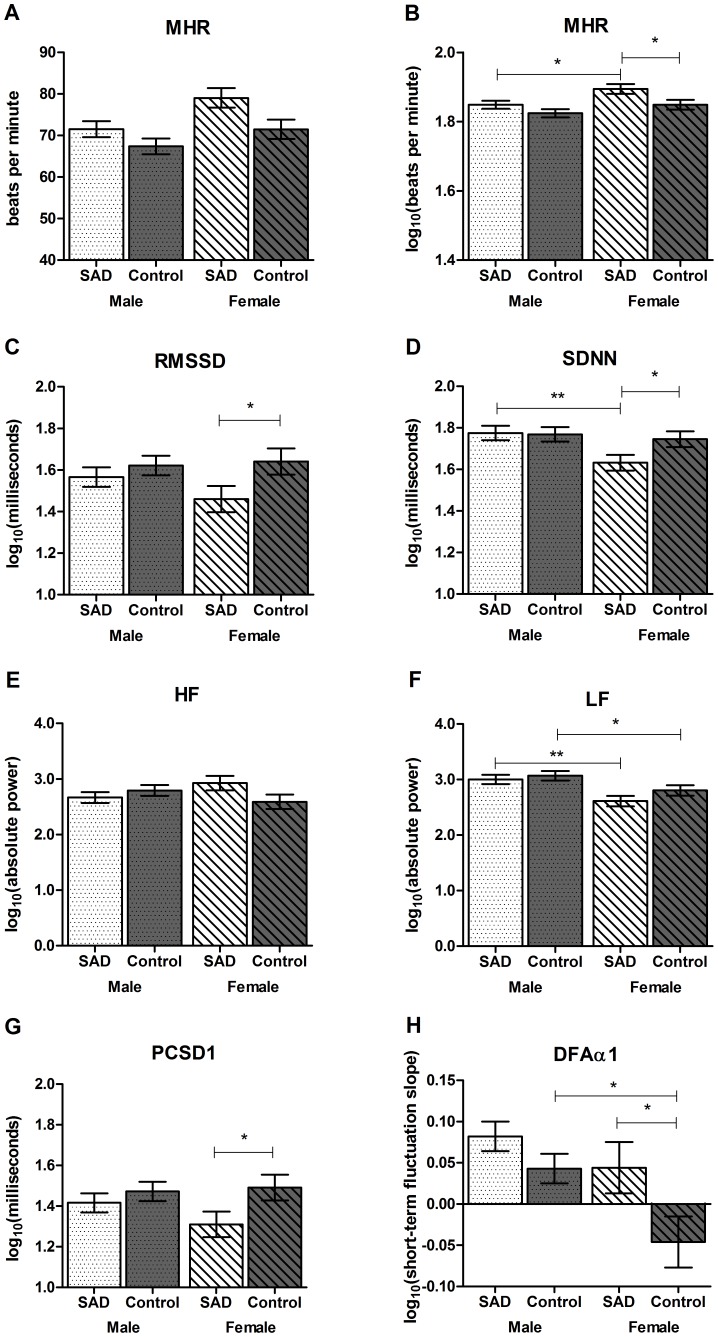
Comparison of resting heart rate variability between males and females with social anxiety, compared to controls, across mean heart rate (row 1), as well as time (row 2), frequency (row 3), and non-linear (row 4) domains. *Note.* All variables are log-transformed (base 10), except for A (which depicts untransformed beats per minute). Error bars depict standard error of the mean. Significance taken from multivariate analysis of variance with group and gender entered as between subjects variables. Significance tests were not conducted on untransformed mean heart rate. MHR = mean heart rate, beats/min, SDNN = standard deviation of all R-R intervals, RMSSD = square root of mean squared differences of successive R-R intervals, LF = low frequency, HF = high frequency, PCSD1 =  standard deviation of the Poincaré plot perpendicular to the line of identity, DFAα1 =  detrended fluctuation analysis of the short-fluctuation slope. *p<.05, **p<.01.

Between the SAD and control groups, significant reductions were observed for RMSSD, HF, and PCSD1, and significant increases in DFAα1 and mean heart rate; see Table 2. Averaged across groups, females exhibited increased heart rate (*F*(1, 102) = 7.34, *p* = .008, *d* = .53) and DFAα1 (*F*(1, 102) = 7.11, *p* = .009, *d* = .50), with decreased SDNN (*F*(1, 102) = 4.98, *p* = .028, *d* = .44) and LF (*F*(1, 102) = 12.89, *p* = .001, *d* = .72); see Figure 1 (also see Table S1 for untransformed means and standard deviations of heart rate variables).

**Table 2:** Log–transformed HRV between groups.

	SAD	Control	*p* value*F*(1, 104)	Cohen’s d
**MHR**	1.87 (0.07)	1.83 (0.06)	.009	.62
**SDNN**	1.72 (0.19)	1.76 (0.20)	.153	.21
**RMSSD**	1.52 (0.27)	1.63 (0.27)	.032	.41
**HF**	2.64 (0.55)	2.85 (0.59)	.044	.37
**LF**	2.84 (0.50)	2.96 (0.47)	.160	.25
**PCSD1**	1.37 (0.27)	1.48 (0.27)	.032	.41
**DFAα1**	0.07 (0.12)	0.01 (0.13)	.008	.48

*Note.* Variables are log-transformed (base 10) and depict means (standard deviations). Significance values taken from multivariate analysis of variance with group and gender entered as between subjects variables. MHR = mean heart rate, beats/min, SDNN = standard deviation of all R-R intervals, RMSSD = square root of mean squared differences of successive R-R intervals, LF = low frequency, HF = high frequency, PCSD1 =  standard deviation of the Poincaré plot perpendicular to the line of identity, DFAα1 =  detrended fluctuation analysis of the short-fluctuation slope.

Post-hoc MANOVAs split by gender indicated that, compared to females in the control group, females with SAD exhibited significantly lower SDNN (*F*(1, 40) = 4.37, *p* = .043, *d* = .64), RMSSD (*F*(1, 40) = 4.14, *p* = .049, *d* = .63), PCSD1 (*F*(1, 40) = 4.14, *p* = .049, *d* = .63), and significantly higher mean heart rate (*F*(1, 40) = 5.34, *p* = .026, *d* = .71) and DFAα1 (*F*(1, 40) = 4.15, *p* = .048, *d* = .63). A trend was exhibited for reduced HF in females with SAD compared to female controls (*F*(1, 40) = 3.28, *p* = .078, *d* = .63). There were no significant differences on any HRV measure between males in the SAD and control groups, smallest *p*-value = .13. As can be seen in Figure 1, reductions in HRV were observed in females with social anxiety compared to females in the control group.

### Associations between Medication, Comorbidity, and HRV

There were no significant correlations observed between age, BMI, and any HRV measure within the SAD group, all *p*-values >.05. Number of comorbid disorders also did not correlate with any HRV measure. Participants using psychotropic medications were taking between one and three medications on the day of physiological data recording (*n* = 30), including antidepressants (*n* = 28), mood stabilizers (*n* = 11), antipsychotics (*n* = 5), and benzodiazepines (*n* = 2). Compared to those participants who were not medicated, medicated SAD participants exhibited significant reductions in SDNN (*F*(1, 51) = 7.02, *p* = .01, *d* = .73), RMSSD (*F*(1, 51) = 6.79, *p* = .01, *d* = .72), and PCSD1 (*F*(1, 51) = 6.80, *p* = .01, *d* = .72), with significant increases in mean heart rate (*F*(1, 51) = 8.96, *p* = .004, *d* = .82), and DFAα1 (*F*(1, 51) = 3.99, *p* = .05, *d* = .54). Independent samples *t*-tests indicated that medicated participants reported significantly higher levels of harmful drinking (AUDIT, *t*(37.59) = 2.23, *p* = .02, *d* = .65), and greater anxiety (DASS-A, *t*(51) = 2.03, *p* = .05, *d* = .57), with no other significant differences in symptom measures between groups. Chi-squared analysis revealed a borderline significant difference in the proportion of females medicated (71.4%) compared to males (43.8%) (Χ^2^ (1) = 3.92, *p* = .05). There were also a greater proportion of comorbid medicated participants (96.6%) compared to those that were not medicated (66.7%) (Χ^2^ (1) = 8.32, *p* = .004).

### HRV and Symptom Severity

Bivariate correlations examined relationships between HRV and symptom severity, see Table 3. In particular, reduced HRV (notably, SDNN, RMSSD, and PCSD1) was associated with increasing self-reported symptom severity as measured by the SPAI, DASS-A and DASS-S, LSAS, and SIAS. These significant correlations were associated with a moderate effect size [28]. Additional significant moderate effects were found between HRV and increasing harmful alcohol use, particularly for increases in mean heart rate and DFAα1. Lastly, increased psychological distress, using the k10, was moderately associated with reduced HRV (*r* coefficients between −.32 and −.37).

**Table 3:** Bivariate correlation coefficients between HRV and symptom severity measures.

	SPAI^a^	DASS-D	DASS-A	DASS-S	LSAS^b^	SIAS^c^	SPS^c^	AUDIT^d^	K10^c^
**MHR**	.15	.02	.32*	.16	.13	.13	.15	.44**	.21
**SDNN**	−.30*	−.27*	−.26	−.07	−.32*	−.35*	−.28	−.28*	−.37**
**RMSSD**	−.30*	−.34*	−.32*	−.13	−.37**	−.38**	−.25	−.34*	−.32*
**HF**	.18	−.25	−.26	−.12	−.27	−.35*	−.17	−.23	−.22
**LF**	−.24	−.23	−.18	−.05	−.27	−.32*	−.19	−.08	−.34*
**PCSD1**	−.30*	−.34*	−.32*	−.13	−.37**	−.38*	−.25	−.34*	−.32*
**DFAα1**	−.09	.22	.27*	.22	.15	.10	.07	.37**	.11

*Note*. HRV measures are log transformed. SPAI = Social Phobia Anxiety Inventory, 23-item version; DASS = Depression, Anxiety, and Stress Scale; LSAS = Liebowitz Social Anxiety Scale; SIAS = Social Interaction Anxiety Scale; SPS = Social Phobia Scale; AUDIT = Alcohol Use Disorders Identification Test; k10 =  Kessler psychological distress scale, 10-items.

* *p*<.05,

** *p*<.01 (two-tailed).

a *n* = 51;

b *n* = 50;

c *n* = 49;

d *n* = 52.

## Discussion

In the present study, we provide evidence that patients diagnosed with social anxiety disorder exhibit reduced HRV in comparison to controls. These reductions were associated with small to moderate effect sizes. Although no significant interaction was found between group and gender, we observed a specific reduction across a number of HRV measures for females with social anxiety disorder, compared to controls. Most importantly, we found that autonomic dysfunction was associated with increasing levels of social anxiety symptoms, as well as psychological distress and alcohol use. Of note, these significant associations were only found for social anxiety, but not depressive, symptoms. Polyvagal theory predicts that variability in vagal tone reflects the capacity for social engagement [7]. In line with this, the present study demonstrates that social interaction anxiety was related to reduced HRV. Greater reductions in HRV in the clinical group were associated with increasing severity of social interaction anxiety, fear, and avoidance.

We also conducted analysis to explore the influence of gender on HRV. Previous evidence suggests that females exhibit greater parasympathetic activity at rest, with greater sympathetic activity found in males (reviewed in [14], [15]). Consistent with previous findings [10], [36], [38], we found that females, on average, exhibited higher resting mean heart rate and reductions across a number of HRV measures. However, the specific finding that females with social anxiety displayed reductions in HRV, compared to female controls, may indicate a greater sensitivity to the effects of social anxiety on parasympathetic nervous system reactivity. Interestingly, this difference was not observed in males with social anxiety, compared to controls, nor were there any significant interactions between group and gender. More pronounced reductions in HRV have also been previously observed in females with social anxiety, although this was in a geriatric sample [11]. Some neural evidence suggests that there may be gender differences when examining associations between HRV and functional activity in particular neural regions within social anxiety [39]. The results from the present study extend upon such evidence to suggest that females with social anxiety exhibit reductions in resting HRV that may imply an increased greater cardiovascular risk in this population.

Although a number of explanations may exist for reduced HRV in the medicated SAD participants, results suggest that this group exhibited a higher proportion of comorbid disorders and higher alcohol use. This finding, in combination with our previous work [40], [41] may suggest that the reductions in HRV are due to the combined effects of comorbidity and harmful alcohol use, rather than the effects of medication per se. Given that anxiety disorders are highly comorbid with depression [4], there may be a shared similarity across these comorbid disorders in reduced HRV that may also underlie a common risk factor for CVD. Further, alcohol consumption reduces neural control over cardiac activity, which acts to reduce HRV; repeated activation of this network through increasing harmful alcohol use may lead to chronic reductions in HRV [7]. As social anxiety is associated with significant comorbidity with alcohol use disorders [42], a lack of autonomic flexibility in medicated social anxiety patients may reflect underlying increased anxiety associated with social interaction and chronic alcohol use.

The significant associations we observed between reduced HRV and increased symptom severity have implications for the potential use of HRV as a marker of treatment response. For example, increases in HRV have been associated with positive response to cognitive treatments in panic disorder, with benefits over antidepressants [43], [44]. It would therefore be of interest to examine whether changes in social anxiety symptoms may also be reflected in greater reductions in HRV. A recently published study found that reductions in self-reported social anxiety due to psychological treatment were best predicted by change in cardiac vagal tone over time [13]. Thus parasympathetic reactivity may help to predict treatment responders in social anxiety disorder. Further, targeted interventions aimed at enhancing HRV may also impact upon anxiety symptoms. For example, Zen meditation [45] and slow breathing [46] can increase HRV in anxious populations. As anxiety is associated with increased mortality and risk for CVD [1], [47], any psychological or pharmacological intervention that successfully modulates vagal tone may also result in longer-term cardioprotective effects. Thus, we intend to investigate in future studies whether successful treatment in social anxiety may be indexed by increased HRV, as well as whether HRV may represent a predictive marker for those who respond best to treatment.

There are a number of limitations to the interpretation of the results from the present study. Firstly, the SAD group exhibited a significantly higher BMI than controls. Although mixed evidence exists about the relationship of BMI to HRV, more robust evidence has found associations between age and HRV [36]. Importantly, however, age and BMI did not significantly correlate with any of our HRV measures. Secondly, whilst the SAD group exhibited a high degree of comorbidity and medication use, this pattern was consistent with treatment-seeking participants recruited from the community [48]. A third point pertains to the significant medication use exhibited in female, compared to male, social anxiety participants which may partially explain HRV reductions within this group. Due to limited power in the unmedicated female group (*n* = 6), we were unable to conduct analyses comparing medicated and unmedicated females. Although we believe that use of this sample of patients increases the generalizability of these findings, further research employing larger samples are required to explore the influence of gender, comorbidity, and medication use on cardiac control in social anxiety. Lastly, a number of studies examining differences in resting-state HRV between groups have argued for the need to statistically control for factors such as smoking use, levels of physical activity, age, and other factors associated with differences in HRV (e.g. [10]). As we have previously argued [49], these studies suffer from an erroneous application of ANCOVA. ANCOVA is a commonly used analytic procedure in psychiatry research to statistically covary for differences between groups. However, as these groups are not randomly assigned, the covariate(s) are likely to share variance with the group factor and thus the association between the dependent variable and group can be reversed, reduced, or enhanced by the addition of one or more covariates. As such, the use of such an analytic strategy would only further complicate interpretation of the above analyses.

In conclusion, the present study demonstrated that social anxiety is characterized by reductions in HRV, with decreased HRV in socially anxious females, and that these reductions are associated with increased social anxiety symptoms. Clinicians may need to consider reducing risk factors associated with CVD in addition to specific anxiety treatment, including modifiable risk factors (for example, changing diet, exercise, or smoking). Further research is now needed to determine whether treatments targeting HRV may positively impact upon social anxiety symptom severity and whether successful treatment of social anxiety or mood symptoms using psychological treatments (for example, Cognitive-Behaviour Therapy) may improve HRV and associated physical health outcomes.

## Supporting Information

Table S1 **Means (and standard deviations) for heart rate variables prior to log-transformation.** *Note.* MHR = mean heart rate, beats/min, SDNN = standard deviation of all R-R intervals, RMSSD = square root of mean squared differences of successive R-R intervals, LF = low frequency, HF = high frequency, PCSD1 =  standard deviation of the Poincaré plot perpendicular to the line of identity, DFAα1 =  detrended fluctuation analysis of the short-fluctuation slope.
(DOC)
