# Supplementary material for: Reduced Heart Rate Variability in Social Anxiety Disorder: Associations with Gender and Symptom Severity
Source: PLoS One. 2013 Jul 30;8(7):e70468. doi: 10.1371/journal.pone.0070468 (PMC3728204; doi:10.1371/journal.pone.0070468)
Supplement: Table S1 — Means (and standard deviations) for heart rate variables prior to log-transformation. Note. MHR = mean heart rate, beats/min, SDNN = standard deviation of all R-R intervals, RMSSD = square root of mean squared differences of successive R-R intervals, LF = low frequency, HF = high frequency, PCSD1 = standard deviation of the Poincaré plot perpendicular to the line of identity, DFAα1 = detrended fluctuation analysis of the short-fluctuation slope. (DOC) [file pone.0070468.s001.doc]

Table S1. Means (and standard deviations) for heart rate variables prior to log-transformation

|  | **Male** | | | **Female** | | |
| --- | --- | --- | --- | --- | --- | --- |
|  | **SAD** | **Control** | **SAD** | | **Control** |  |
| **MHR** | 71.52 (11.99) | 67.39 (9.78) | 79.03 (10.27) | | 71.48 (10.72) |  |
| **SDNN** | 64.73 (26.73) | 65.69 (32.74) | 46.13 (18.70) | | 60.70 (28.11) |  |
| **RMSSD** | 44.07 (27.86) | 49.84 (31.26) | 35.31 (24.56) | | 54.98 (41.66) |  |
| **HF** | 996.47 (1572.62) | 1450.49 (2364.12) | 926.00 (1365.25) | | 2034.30 (3534.34) |  |
| **LF** | 1689.95 (1814.09) | 2033.21 (2206.89) | 684.37 (724.08) | | 864.51 (594.04) |  |
| **PCSD1** | 31.21 (19.73) | 35.30 (22.15) | 25.01 (17.41) | | 38.94 (29.52) |  |
| **DFAα1** | 1.24 (0.27) | 1.13 (0.24) | 1.15 (0.29) | | 0.95 (0.31) |  |

*Note.* MHR = mean heart rate, beats/min, SDNN = standard deviation of all R-R intervals, RMSSD = square root of mean squared differences of successive R-R intervals, LF = low frequency, HF = high frequency, PCSD1 = standard deviation of the Poincaré plot perpendicular to the line of identity, DFAα1 = detrended fluctuation analysis of the short-fluctuation slope.
